# Hispines (Chrysomelidae, Cassidinae) of La Selva Biological Station, Costa Rica

**DOI:** 10.3897/zookeys.157.1338

**Published:** 2011-12-21

**Authors:** Charles L. Staines

**Affiliations:** 1 Department of Entomology, MRC 187, National Museum of Natural History, Smithsonian Institution, P. O. Box 37012, Washington, DC 20013-7012, United States

**Keywords:** Chrysomelidae, hispine, La Selva Biological Station

## Abstract

Survey work from 1992–2001 identified 139 species of hispines at the lowland part of La Selva Biological Station, Costa Rica. The tribe Cephaloleiini was the most speciose with 58 species (41.7%) followed by the Chalepini with 55 (39.5%). The fauna is most closely related to that in South America but with some genera which are more speciose in the Nearctic Region. Plant associations are known for 88 (63.3%) of the species but many of these are merely collecting records, not host plant associations. The first plant associations are reported for *Alurnus ornatus*, *Alurnus salvini*, and *Acentroptera nevermanni*.

## Introduction

Hispines comprise half of the subfamily Cassidinae (sensu lato) in the family Chrysomelidae within the order Coleoptera (Staines 2002b). Until recently, most authors treated the group as a separate subfamily but recent work has shown that there is no biological or morphological reason to retain sub-familial status (Staines 2002b). The combined subfamily consists of 6000 species placed in 42 tribes (Staines 2002b). See Staines (2002b) for a detailed history of the classification of the two groups.

The combination of the Hispinae with the Cassidinae (s. str.) has created difficulty in having a handy term to use for these beetles. Several have been proposed but they are cumbersome. Until an easily used term is coined for this group, I continue to use “hispines” in the traditional sense of the genera and species in the former subfamily Hispinae (see Seeno and Wilcox 1982 for a list of genera).

The adult hispine head is opisthognathous, prominent, visible from above, at least to behind the eyes. The frons is prominent, exposed or rarely retracted. The antennae are not retractable and are closely inserted between the eyes. The pronotum is narrower than the elytra; it is more or less quadrangular or trapezoidal, with definite anterior angles which may have a small tubercle. The scutellum is always visible. The elytra lack lateral expansions or have reduced and discontinuous expansions. The margins are usually denticulate or with spines.Larvae are either leaf-miners or free living. They have eight pairs of abdominal spiracles which are well developed and dorsally placed; with the eighth abdominal segment terminal, and with free hind margin (Staines 2002b, 2006).

Ecologically, New World hispines fall into three feeding groups: external feeders; sheath, appressed or rolled-leaf feeders; and leaf-miners. In the Old World, some species have been reported as stem borers in herbaceous or semi-ligneous plants, but this has not been reported from the New World. The biology of few species has been studied; most are not associated with a host plant or plant family.

## Methods

### Study area

La Selva Biological Station (10°26'N, 83°59'W) is located in the Atlantic tropical lowlands of Costa Rica and is adjacent to Braulio Carrillo National Park. It is about 100 km from San José. The station comprises 1600 hectares. Habitat is a mosaic of primary forest, early secondary pasture, young secondary forest, abandoned plantations, and selectively logged primary forest. The elevation varies from 35 to 137 m. The station is near the confluence of Rio Puerto Viejo and Rio Sarapiquí. It is owned and operated by the Organization for Tropical Studies (McDade and Hartshorn 1994).

Rainfall varies from 152.0 mm (March) to 480.7 mm (July) with a total 4 m per year. The dry season is short and not severe (Sanford et al. 1994).

There are 1744 plant species documented from La Selva. The most speciose families are Pteridophyta, Orchidaceae, Araceae, Rubiaceae, Melastomataceae, Fabaceae, and Piperace (Hartshorn and Himmel 1994).

The Arthropods of La Selva (ALAS) project was started in 1991 (http://viceroy.eeb.uconn.edu/ALAS/ALAS.html). An existing building on the station was remodeled as an entomology laboratory and four technicians were trained in the National Biodiversity Institute (INBio) six-month parataxonomist course. From 1992 until 2000 the project was funded by separate grants from the U.S. National Science Foundation (Biotic Surveys and Inventories Program). From 2001–2006 the focus of the project shifted to a transect survey from La Selva Biological Station to the summit of Volcan Barva. This paper deals only with the results at the La Selva Biological Station.

Daily operations of ALAS were conducted by the parataxonmists under the direction of the principal investigators John T. Longino, Evergreen State College, and Robert K. Colwell, University of Connecticut. Over the course of the project there were over 100 collaborating taxonomists.

### Survey aethods

The ALAS survey consisted of both structured and directed sampling. Structured sampling consisted of black-lights, Malaise and flight intercept traps, and canopy fogging (see Furth et al. 2003 for summary).

Passive black-lights were utilized from 1993 to 1999 at twelve sites, six on the ground and six in the canopy. Malaise traps were used at sixteen sites from 1993 to 2000. Specimens were collected directly into ethanol and the traps emptied every two weeks. Flight intercept traps were place at sixteen sites and samples were collected every two weeks.

Canopy fogging was conducted in 1993–1994, 1996, and 2000. Sixteen trees were fogged: six trees of the most common species at La Selva, six trees of an intermediate abundant species, and trees of six different species. The tree selected had large crowns with little crown overlap and with good climbing access.

Directed collecting for chrysomelids used beating, sweeping, visual observation, known host plant observation, and use of a mid-canopy shaker net.

## Results and discussion

### Species richness at La Selva

As of the end on 2001, a total of 139 hispine species have been collected at La Selva Biological Station (see Table 1).

Quantitative inventory by non-specialists using standard sampling techniques can capture about half of the fauna. Individual methods are needed to sample the rest of the community. Sweeping, beating, and host plant sampling are the best methods. Fogging, Berlese funnels, and Malaise traps capture a few species usually not otherwise collected but are not sufficient in themselves to indicate the actual fauna.

**Table 1.** Hispines known from La Selva Biological Station and their plant associations (**A**=adult plant feeding; **L**=larval host plant; **U**=unspecified).

**Tribe**	**Genus/Species**	**Plant association**	**Plant family**	**Observation**	**References**
Alurnini	*Alurnus ornatus* Baly	*Chamaedorea* sp.	Arecaceae	A	New observation
Alurnini	*Alurnus salvini* Baly	*Chamaedorea* sp.	Arecaceae	A	New observation
Arescini	*Chelobasis bicolor* Gray	*Heliconia* sp., *Heliconia latispatha* Benth., *Heliconia tortuosa* Griggs, *Heliconia cathaeta* R. R. Smith	Heliconiaceae	L	Maulik 1937;<br/> Strong 1977a, 1983;<br/> Meskins et al. 2008
		*Musa* sp.	Musaceae	L	
		*Calathea latifolia* Klotzsch	Marantaceae	L	
Arescini	*Chelobasis perplexa* Baly	*Heliconia imbricata* (Kuntze) Baker, *Heliconia latispatha*, *Heliconia pogonantha* Cuford.,	Heliconiaceae	L	Maulik 1932;<br/> Strong and Wang 1977;<br/> McKenna and Farrell 2005; Meskins et al. 2008
		*Heliconia irrasa* R. R. Smith, *Heliconia mariae* Hook.*Calathea insignis* Hort. & Bull.	Marantaceae	L	
Cephaloleiini	*Aslamidium impurum* (Boheman)	*Calathea ovata* Lindl., *Calathea virginalis* Linden, *Calathea insignis*, *Calathea micans* (Mathieru) Koern.	Marantaceae	A	Bondar 1940; Spaeth 1938;
		*Heliconia* sp.	Heliconiaceae	A	Windsor et al. 1992
Cephaloleiini	*Cephaloleia aequilata* Uhmann	Unknown			
Cephaloleiini	*Cephaloleia atriceps* Pic	Unknown			
Cephaloleiini	*Cephaloleia bella* Baly	*Heliconia imbricata*	Heliconiaceae	A	Staines 1996
Cephaloleiini	*Cephaloleia belti* Baly	*Calathea insignis*, *Calathea latifolia* Klotzsch, *Calathea lutea* (Aubl.) GFW Mey., *Ischnosiphon pruinosus* Peterson	Marantaceae	A	Uhmann 1930; Maulik 1932; Strong 1977b, 1982a; Meskins et al. 2008; Descampe et al. 2008; García-Robledo et al. 2010
		*Heliconia imbricata*, *Heliconia latispatha*, *Heliconia pogonantha*, *Heliconia mariae*, *Heliconia tortuosa*, *Heliconia catheta*, *Heliconia irrasa*, *Heliconia vaginalis* Benth., *Heliconia wagneriana* Peterson	Heliconiaceae	A, L	
Cephaloleiini	*Cephaloleia championi* Baly	*Heliconia* sp.	Heliconiaceae	A	Staines 1996
Cephaloleiini	*Cephaloleia congener* Baly	*Heliconia latispatha*, *Heliconia tortuosa*	Heliconiaceae	A	Staines 1996
Cephaloleiini	*Cephaloleia consanguinea* Baly	Unknown			
Cephaloleiini	*Cephaloleia costaricensis* Uhmann	*Chusquea simpliciflora* Munro	Poaceae	U	Meskins et al. 2008
Cephaloleiini	*Cephaloleia deficiens* Uhmann	Unknown			
Cephaloleiini	*Cephaloleia dilaticollis* Baly	*Calathea insignis*, *Calathea lutea*, *Calathea inocephala* (Kuntze), H. Kennedy, *Ischnosiphon pruinosus*	Marantaceae	A	Staines 1996; McKenna and Farrell 2005; Meskins et al. 2008; Descampe et al. 2008; García-Robledo et al. 2010
		*Renealmia* sp., *Renealmia alpinia* (Rottb.) Maas	Zingiberaceae	A, L	
Cephaloleiini	*Cephaloleia disjuncta* Staines	*Vitex copperi* Stanley	Verbenaceae	A	Staines 1998
Cephaloleiini	*Cephaloleia distincta* Baly	*Calathea* sp.	Marantaceae	A	Staines 1996
		*Heliconia imbricata*	Heliconiaceae	A	
Cephaloleiini	*Cephaloleia dorsalis* Baly	*Costus* sp., *Costus pulverulentus* C. Presl., *Costus malortieanus* Wendl., *Costus larvis* Ruiz. & Pav.	Costaceae	A, L	Staines 1996; McKenna and Farrell 2005; Meskins et al. 2008; García-Robledo and Horvitz 2009; García-Robledo et al. 2010
		*Renealmia* sp.	Zingiberaceae	A	
Cephaloleiini	*Cephaloleia elegantula* Baly	Unknown			
Cephaloleiini	*Cephaloleia erichsonii* Baly	*Calathea gymnocarpa* H. Kennedy, *Calathea inocephala*, *Calathea leucostachys* Hook., *Calathea insignis*, *Calathea latifolia*, *Calathea lutea*	Marantaceae	A	Staines 1996; Strong 1977a; McKenna and Farrell 2005; Meskins et al. 2008; Descampe et al. 2008
		*Heliconia* sp., *Heliconia catheta*, *Heliconia latispatha*, *Heliconia mariae*, *Heliconia vaginalis*, *Heliconia wagneriana*	Heliconiaceae	A	
Cephaloleiini	*Cephaloleia exigua* Uhmann	Unknown			
Cephaloleiini	*Cephaloleia fenestrata* Weise	*Ischnosiphon* sp., *Ischnosiphon cerotus* Leos., *Pleiostachya pruinosa* K. Schum.	Marantaceae	L	Staines 1996; Strong 1977a; Johnson 2004a
Cephaloleiini	*Cephaloleia flava* Uhmann	Unknown			
Cephaloleiini	*Cephaloleia fulvolimbata* Baly	Unknown			
Cephaloleiini	*Cephaloleia heliconicae* Uhmann	*Heliconia* sp.	Heliconiaceae	A	Staines 1996
		*Calathea insignis*	Marantaceae	S	
Cephaloleiini	*Cephaloleia histrionica* Baly	Unknown			
Cephaloleiini	*Cephaloleia lata* Baly	*Chamaedorea tepejilote* Liebm., *Chamaedorea wendlandiana* Hemsl.	Arecaceae	A	McKenna and Farrell 2005; Meskins et al. 2008
Cephaloleiini	*Cephaloleia mauliki* Uhmann	*Heliconia* sp.	Heliconiaceae	A	Uhmann, 1930; Maulik 1932, 1937
		*Calathea insignis*	Marantaceae	A	
Cephaloleiini	*Cephaloleia metallescens* Baly	*Bactris major* Jacq., *Chamaedorea wendlandiana*	Arecaceae	U	Meskins et al. 2008
Cephaloleiini	*Cephaloleia nevermanni* Uhmann	*Calathea insignis*, *Calathea macrosepala* K. Schumann	Marantaceae	A	Uhmann 1930; Staines 1996
		*Heliconia imbracata*	Heliconiaceae	A	
Cephaloleiini	*Cephaloleia nigricornis* (Fabricius)	Unknown			
Cephaloleiini	*Cephaloleia ornatrix* Donckier	*Heliconia* sp.	Heliconiaceae	A	Strong 1977a
Cephaloleiini	*Cephaloleia placida* Baly	*Renealmia* sp., *Renealmia alpinia* (Rottb.) Maas	Zingiberaceae	A	Staines 1996; García-Robledo and Horvitz 2009; García-Robledo et al. 2010
Cephaloleiini	*Cephaloleia puncticollis* Baly	*Calathea insignis*	Marantaceae	L	Uhmann 1930; Seifert and Seifert 1976; Staines 1996
		*Heliconia imbricata*, *Heliconia latispatha*	Heliconiaceae	L	
		*Musa* sp.	Musaceae	L	
Cephaloleiini	*Cephaloleia quadrilineata* Baly	*Heliconia imbricata*, *Heliconia latispatha*	Heliconiaceae	A	Staines 1996
Cephaloleiini	*Cephaloleia reventazonica* Uhmann	*Heliconia latispatha*	Heliconiaceae	A	Staines 1996
Cephaloleiini	*Cephaloleia ruficollis* Baly	Unknown			
Cephaloleiini	*Cephaloleia sallei* Baly	*Heliconia* sp., *Heliconia irrasa*, *Heliconia catheta*, *Heliconia latispatha*, *Heliconia mariae*, *Heliconia vaginalis*	Heliconiaceae	L	Strong 1977a; Staines 2004a; McKenna and Farrell 2005; Meskins et al. 2008; Descampe et al. 2008
		*Renealmia strobilifera*			
		*Calathea inocephala*, *Calathea latifolia*, *Calathea lutea*,	Zingiberaceae	A	
		*Ishnosiphon pruinosus*	Marantaceae	A	
Cephaloleiini	*Cephaloleia semivittata* Baly	*Calathea marantifolia* Standley	Marantaceae	A	Staines 1996
Cephaloleiini	*Cephaloleia splendida* Staines	Unknown			
Cephaloleiini	*Cephaloleia stevensi* Baly	*Heliconia* sp.	Heliconiaceae	A	Staines 1996; McKenna and Farrell 2005; Meskins et al. 2008
		*Calathea micans*, *Calathea inocephala*, *Calathea latifolia*, *Ishnosiphon pruinosus*	Marantaceae	A	
Cephaloleiini	*Cephaloleia sulciceps* Baly	Unknown			
Cephaloleiini	*Cephaloleia suturalis* Baly	*Costus malorticenus* H. Wendl., *Costus* sp., *Costus pulverulentus*	Costaceae	A	Uhmann 1930; Maulik 1937; Meskins et al. 2008
Cephaloleiini	*Cephaloleia tenella* Baly	Unidentified	Areaceae	A	Staines 1996
Cephaloleiini	*Cephaloleia trimaculata* Baly	ginger lily, *Renealmia* sp.	Zingiberaceae	A	Uhmann 1950; McKenna and Farrell 2005; Meskins et al. 2008
		*Costus pulverulentus*	Costaceae	A	
Cephaloleiini	*Cephaloleia trivittata* Baly	*Calathea haamelii* H. Kennedy, *Calathea macrosepala*	Marantaceae	A	Staines 1996
Cephaloleiini	*Cephaloleia vicina* Baly	*Heliconia* spp., *Heliconia latispatha*, *Heliconia imbricata*	Heliconiaceae	A	Strong 1977a, 1977b, 1981
		*Calathea* spp., *Ischnospihon* spp.	Marantaceae	A	
Cephaloleiini	*Cephaloleia* sp. 1	Unknown			
Cephaloleiini	*Demotispa nevermanni* Uhmann	Unknown			
Cephaloleiini	*Demotispa strandi* Uhmann	*Spermacoce* sp.	Rubiaceae	U	Flowers and Janzen 1997; Staines 2006a
		*Calathea* sp.	Marantaceae	A	
Cephaloleiini	*Demotispa* sp. 1	Unknown			
Cephaloleiini	*Demotispa* sp. 2	Unknown			
Cephaloleiini	*Homalispa gracilis* Baly	Unknown			
Cephaloleiini	*Homalispa nevernmanni* Uhmann	*Oenocarpus panamanus* Bailey	Arecaceae	U	Meskins et al. 2008
Cephaloleiini	*Homalispa* sp. 1	Unknown			
Cephaloleiini	*Imatidium rufiventre* Boheman	*Inga marginata* Willd.	Fabaceae	A	Gilbert et al. 2001
Cephaloleiini	*Imatidium thoracicum* Fabricius	*Calathea insignis*, *Calathea ovata*, *Calathea virginalis*, *Calathea lutena*	Marantaceae	A	Spaeth 1938; Bondar 1940; Windsor et al. 1992; Meskins et al. 2008
		*Heliconia latispatha*, *Heliconia catheta*, *Heliconia irrasa*, *Heliconia wagneriana*	Heliconiaceae	A	
Cephaloleiini	*Solenispa leptomorpha* (Baly)	Unknown			
Cephaloleiini	*Stenispa graminicola* Uhmann	Unknown			
Cephaloleiini	*Stenispa sallei* Baly	Unknown			
Cephaloleiini	*Stenispa vespertina* Baly	*Cyperus* sp.	Cyperaceae	L	Bondar 1931b
Cephaloleiini	*Stilpnaspis rubiginosus* (Boheman)	Unknown			
Chalepini	*Anisostena pilatei* (Baly)	Unknown			
Chalepini	*Baliosus productus* (Baly)	Unidentified	Bignoniaceae	L	Hespenheide and Dang 1999
Chalepini	*Baliosus* sp.1	*Urera bogataense* ?	Urticaceae	L	Hespenheide and Dang 1999
Chalepini	*Baliosus* sp. 2	Unknown			
Chalepini	*Carinispa nevermanni* Uhmann	*Malpighia glabra* L., *Bunchosia* sp.	Malpighiaceae	L	Uhmann 1934; Flowers and Janzen 1997
Chalepini	*Chalepus amiculus* Baly	Unknown			
Chalepini	*Chalepus angulosus* Baly	Unknown			
Chalepini	*Chalepus assmani* Uhmann	Unknown			
Chalepini	*Chalepus bellulus* (Chapuis)	*Digitaria eriantha* Steud., *Oryza* sp.	Poaceae	L	Maes and Staines 1991; Staines 1997; Flowers and Janzen 1997
		Unidentified	Arecaceae	U	
		*Phaseolus* sp.	Fabaceae	U	
Chalepini	*Chalepus brevicornis* (Baly)	Unknown			
Chalepini	*Chalepus consanguineus* (Baly)	*Lasiacis* sp. Unidentified	Poaceae	L	Uhmann 1935; Hespenheide and Dang 1999
Chalepini	*Chalepus digressus* Baly	*Lasiacis* sp.	Poaceae	L	Memmott et al. 1993
Chalepini	*Chalepus nigripictus* Baly	Unknown			
Chalepini	*Chalepus pici* Descarpentries & Villiers	Unknown			
Chalepini	*Chalepus similatus* Baly	Unknown			
Chalepini	*Chalepus tappesi* Chapuis	Unknown			
Chalepini	*Chalepus verticalis* (Chapuis)	*Phaseolus* sp.	Fabaceae	U	Maes and Staines 1991
Chalepini	*Chalepus* sp. 1	Unknown			
Chalepini	*Chalepus* sp. 2	Unknown			
Chalepini	*Charistena ruficollis* (Fabricius)	*Zea mays* L., *Paspalum conjugatum* Berg	Poaceae	U	Bondar 1931a; Schlottfeldt 1944; Maulik 1937; Maes and Staines 1991
		*Glycine max* (L.) Merr.	Fabaceae	U	
		*Coffea* sp.	Rubiaceae	U	
Chalepini	*Euprionota aterrima* Guérin-Méneville	Unknown			
Chalepini	*Glyphuroplata nigella* Weise	*Valota* sp., *Eriochloa gracilis* (Fourn.) Hitchc.	Poaceae	L	Riley 1985; Hespenheide and Dang 1999
		*Mimosa* sp.	Fabaceae	U	
Chalepini	*Heptispa limbata* (Baly)	*Cassia grandis* L., *Cassia fruitcosa* Mill., *Inga* sp., *Machaerium* sp.	Fabaceae	L	Uhmann 1934, 1937; Memmott et al. 1994; Hespenheide and Dang 1999
		*Serjania* sp.	Sapindaceae	U	
		*Olyra latifolia*	Poaceae	U	
Chalepini	*Heterispa vinula* (Erichson)	*Triumfetta josefina* Polak,	Tilaceae	L	Uhmann 1934, 1937; Maulik 1937; Hespenheide and Dang 1999; Casari and Teizeira 2004
		*Apeiba membranacea* Spruce ex. Benth.			
		*Guazuma ulmifolia* L.	Sterculiaceae	L	
		*Sida* sp. *Sida rhombifolia* L., *Sida carpinifolia* K. Schum.	Malvaceae	L	
		*Infigofera* sp.	Fabaceae	L	
Chalepini	*Octhsipa bimaculata* Uhmann	*Stigmaphyllum lindenianum* A. Juss.	Malphigiaceae	L	Hespenheide and Dang 1999
Chalepini	*Octhispa decepta* (Baly)	*Stigmaphyllum lindenianum*	Malphigiaceae	L	Hespenheide and Dang 1999
Chalepini	*Octhispa elegantula* Baly	*Serjania* sp., *Paullinia* sp.	Sapindaceae	L	Uhmann 1937; Hespenheide and Dang 1999
		*Pithecoctenium echinatum* K. Schum.	Bignoniaceae	U	
Chalepini	*Octhispa elevata* (Baly)	*Paullinia* sp.	Sapindaceae	L	Uhmann 1934; Maulik 1937; Hespenheide and Dang 1999
		*Pithecoctenium echinatum*	Bignoniaceae	U	
Chalepini	*Octhispa haematopyga* Baly	*Colubrina spinosa* Don. Sm.	Rhamnaceae	L	Hespenheide and Dang 1999
Chalepini	*Octhispa nevermanni* Uhmann	*Ochroma lagopus* Rowlee	Bombaceae	L	Hespenheide and Dang 1999
Chalepini	*Oxychalepus alienus* (Baly)	*Centrosema macrocarpum* Benth., *Cassia fruticosa*	Fabaceae	L	Flowers and Janzen 1997; Hespenheide and Dang 1999
Chalepini	*Oxychalepus posticatus* (Baly)	*Cassia oxyphylla* Kunth., *Cassia hayesiana* Standl., *Cassia fruticosa*	Fabaceae	L	Uhmann 1937; Memmott et al. 1994; Hespenheide and Dang 1999
Chalepini	*Oxyroplata nr. bellicosa* Uhmann	*Bamosteroa argentea* Spreng.	Malphighiaceae	L	Uhmann 1937
Chalepini	*Pentispa explanata* (Chapuis)	*Pithecoctenium* sp.	Bignoniaceae	L	Uhmann 1934
Chalepini	*Pentispa fairmairei* (Chapuis)	*Chusquea* sp.	Poaceae	U	Uhmann 1937; Maulik 1937; Morris et al. 2004
		*Calea urticaefolia* (P. Mill.) DC, *Calea axillaries* DC., *Vernonia mollis* H.B.K., *Verbesina* sp., *Eupatorium populifolium* Hook. & Arn., *Clibadium* sp., *Lepidaploa tortuosa* (L.) H. Rob.	Asteraceae	L	
		*Elephantopus spicatus* Aubl., *Malpighia glabra*	Malpighiaceae	U	
		*Serjania* sp.	Sapindaceae	U	
Chalepini	*Pentispa* sp. 1	Unknown			
Chalepini	*Pentispa* sp. 2	Unknown			
Chalepini	*Platocthispa championi* (Baly)	*Piper* sp.	Piperaceae	L	Hespenheide and Dang 1999
Chalepini	*Platocthispa emorsitans* (Baly)	*Calathea* sp., *Calathea insignis*,	Marantaceae	U	Staines 2004a; Meskins et al. 2008
		*Calathea latifolia*			
		*Costus* sp.	Costaceae	U	
		*Heliconia catheta*, *Heliconia irrasa*, *Heliconia latispatha*	Heliconiaceae	U	
Chalepini	*Platocthispa* sp. 1	*Ochroma lagopus*	Bombaceae	L	Hespenheide and Dang 1999
Chalepini	*Probaenia armigera* (Baly)	*Piptocarpha chontalensis* Baker in Mart.	Asteraceae	L	Hespenheide and Dang 1999
Chalepini	*Probaenia pici* Uhmann	*Mikania guaco* Humb. & Bonpl.	Asteraceae	L	Hespenheide and Dang 1999
Chalepini	*Probaenia* sp. 1	*Arrabidaea chica* (Humb. & Bonpl.) Verl.	Bignoniaceae	L	Hespenheide and Dang 1999
Chalepini	*Sumitrosis amica* (Baly)	*Heliconica* sp.	Heliconiaceae	L	Hespenheide and Dang 1999
Chalepini	*Sumitrosis fryi* (Baly)	*Eupatorium populifolium*	Asteraceae	L	Uhmann 1937
Chalepini	*Sumitrosis instabilis* (Baly)	Unknown			
Chalepini	*Sumitrosis pallescens* (Baly)	*Chamaecrista fasciculata* (Michx.) Greene, *Chamaecrista nictitans* (L.) Moench.	Caesalpiniaceae	U	Cavey 1994
Chalepini	*Sumitrosis terminatus* (Baly)	Unidentified	Fabaceae	L	Hespenheide and Dang 1999
Chalepini	*Uroplata fusca* Chapuis	*Pithecactenium echinatum*, *Arrabidaea mollisima* Bureau & K. Schm.	Bignoniaceae	L	Uhmann 1934, 1937; Memmott et al. 1994
		*Malpighia glabra*	Malpighiaceae	U	
Chalepini	*Uroplata sculptilis* Chapuis	*Clibadium aspersum* DC, *Synedrella nodiflora* Gaertn.	Asteraceae	L	Uhmann 1934, 1937; Hespenheide and Dang 1999
		*Inga edalis* Mart.	Fabaceae	L	
		*Gouania adenophora* Pilg.	Rhamnaceae	L	
Chalepini	*Uroplata* sp. 1	Unknown			
Chalepini	*Uroplata* sp. 2	Unknown			
Chalepini	*Xenochalepus amplipennis* (Baly)	Unidentified	Fabaceae	L	Hespenheide and Dang 1999
Chalepini	*Xenochalepus erythroderus* (Chapuis)	*Coussapoa nymphaeifolia* Standl., *Coussapoa villosa* Poepp. & Endl., *Cecropia insignis* Liebm, *Pourouma bicolor* (Standl.) C.C. Berg & E.C. van Heusden	Cecropiaceae	L	Hespenheide and Dang 1999
Chalepini	*Xenochalepus rufithorax* (Baly)	Unknown			
Prosopodontini	*Prosopodonta distincta* (Baly)	Unknown			
Prosopodontini	*Prosopodonta dorsata* (Baly)	*Costus* sp.	Costaceae	U	Uhmann 1930; Meskins et al. 2008
		*Chamaedorea wendlandiana*, *Cryosophila warscewiczii* Bartl., *Oenocarpus panamanus* Bailey	Arecaceae	L	
Sceloenoplini	*Acentroptera strandi* Uhmann	*Pentaclethra macroloba* Kuntze	Fabaceae	U	New observation
Sceloenoplini	*Ocnosispa humerosa* Staines	*Conceveiba pleiostemona* Donn. Smith	Euphorbiaceae	A	Staines 2002a
Sceloenoplini	*Pseudispa fulvolimbata* (Baly)	Unknown			
Sceloenoplini	*Sceloenopla antennata* (Baly)	Unknown			
Sceloenoplini	*Sceloenopla bicolorata* Staines	*Sterculia recordiana papyracea* E. Taylor	Sterculiaceae	A	Staines 2002a
Sceloenoplini	*Sceloenopla bidentata* Staines	Unknown			
Sceloenoplini	*Sceloenopla erudita* (Baly)	*Anthurium* sp.	Araceae	L	Uhmann 1944; Hespenheide and Dang 1999
		*Cupania* sp.	Sapindaceae	L	
Sceloenoplini	*Sceloenopla godmani* (Baly)	*Clusia flava* Planch. & Triana	Clusiaceae	L	Hespenheide and Dang 1999
Sceloenoplini	*Sceloenopla gracilenta* (Baly)	Unknown			
Sceloenoplini	*Sceloenopla lampyridiformis* Staines	Unidentified	Viscaceae	L	Staines 2002a
Sceloenoplini	*Sceloenopla longula* (Baly)	Unidentified	Araceae	L	Hespenheide and Dang 1999
Sceloenoplini	*Sceloenopla lutena* Staines	*Virola koschnyi* Warb.	Myristicaceae	A	Staines 2002a
Sceloenoplini	*Sceloenopla minuta* Staines	Unknown			
Sceloenoplini	*Sceloenopla multistriata* Uhmann	*Virola koschnyi*	Myristicaceae	U	Staines 2002a; Maes 2004
		*Phoradendron* sp.	Loranthaceae	U	
		*Persea americana* P. Mill.	Lauraceae	U	
Sceloenoplini	*Sceloenopla nevermanni* Uhmann	*Anthurium* sp.	Araceae	L	Uhmann 1944; Hespenheide and Dang 1999
		*Cupania* sp.	Sapindaceae	L	
Sceloenoplini	*Sceloenopla nigropicta* Staines	*Virola koschnyi*	Myristicaceae	A	Staines 2002a
Sceloenoplini	*Sceloenopla obscurovittata* (Baly)	*Philodendron radiatum radiatum* Schott, *Monstera tenuis* K. Koch	Araceae	L	Hespenheide and Dang 1999
Sceloenoplini	*Sceloenopla proxima* (Baly)	Unknown			
Sceloenoplini	*Sceloenopla scherzeri* (Baly)	*Davilla nitida* (Vahl) Kubitzki	Dilleniaceae	L	Bondar 1937; Hespenheide and Dang 1999
		*Persea gratissima* Gaertn.	Lauraceae	L	
Sceloenoplini	*Sceloenopla subparallela* (Baly)	Unknown			

### Major lineages

The most recent classification of hispines is by Würmli (1975) and Staines (2002b). There are 24 extant tribes of hispines, of which six have been found at La Selva (see Table 1).

Over 40% of the 139 hispine species and 25% of the genera are in the tribe Cephaloleiini. The Cephaloleiini is a New World tribe of 16 genera and 382 species (Staines 2002b). Over 200 species are in the genus *Cephaloleia* Chevrolat (Uhmann 1957, Staines 1996).

At La Selva *Cephaloleia* is the most speciose genus with 44 species from La Selva. The biology of various *Cephaloleia* species has been studied by Strong (1977a, b, 1982a, 1983), Seifert and Seifert (1976), Strong and Wang (1977), Auerbach and Strong (1981), and Morrison and Strong (1981). Since the only identification aid available to these workers was Baly (1885), which covered less than half of the species known from Central America, some of the published names are not associated with the correct species. However the published information does give valuable data on the general biology and ecology of *Cephaloleia* species. Staines (2004a) attempted to associate the biological data with the correct species. Additional biological work and host plant associations have been done by Johnson (2004a, b), Johnson and Horvitz (2005), McKenna and Farrell (2005), Descampe et al. (2008), Meskins et al. (2008), García-Robledo and Horvitz (2009, in press), and García-Robledo et al. (2010).

*Cephaloleia* eggs are flat, with a thin chorion; hence they are subject to desiccation. Eggs are laid on host surfaces. Oviposition sites vary among beetle species and host plant. The most common oviposition sites are leaf surfaces, petioles of immature leaves or inflorescence bracts. Eggs hatch in 10 to 20 days. Larvae begin feeding immediately upon the part of the plant on which the egg was laid. *Cephaloleia* larvae have a water penny-like appearance. They are flat and well adapted to moving between the wet surfaces of Zingiberales leaves, stems, and flowers. Larvae grow very slowly and go through up to eight molts depending on the size of the species and the part of the plant fed on. During their development, larvae of leaf and stem-feeding species utilize several leaves or even leaves on adjacent plants. Inflorescence-feeding larvae are restricted to a single inflorescence. Larvae of *Cephaloleia* species feed on the plant by dragging their mandibles across the plant surface while they crawl forward. This results in an irregularly shaped feeding scar and a trail of frass. Pupation occurs above ground, usually on the stalk of the host plant and lasts about 20 days. Adult *Cephaloleia* are found in the same habitat as larvae and cause similar feeding damage. Several different *Cephaloleia* species as well as other genera may utilize the same leaf, so larval associations require rearing (Strong 1977a, b, 1982a, 1983; Strong and Wang 1977; Auerbach and Strong 1981; and Morrison and Strong 1981).

Seven other genera of Cephaloleiini containing 14 species are known from La Selva. Most of these species are poorly known and not associated with their host plant.

The tribe Arescini consists of four genera and 17 species from the Neotropics (Staines 2002b). One genus and two species are known from Mesoamerica. None of the genera have been revised and little work has been done on the biology. *Chelobasis bicolor* Gray and *Chelobasis perplexa* Baly are found at La Selva. Strong (1977a, 1983) reported the larval host plants of *Chelobasis bicolor* as *Heliconia latispatha* Benth. and *Heliconia tortuosa* Griggs (Heliconiaceae). Strong (1983) reported on the biology of this species indicating that eggs are laid on wet, tender tissue of the host plant and hatch in about 20 days. Larvae begin feeding in rolled leaves immediately after hatching. Development is slow, requiring at least eight months until pupation. Larvae require more than one leaf-roll to complete development and move from maturing leaf-rolls to more tender ones at night. If they are between leaf-rolls at daylight, they hide between the petiole and stalk until nightfall. Adults are polymorphic (in color and size) and long-lived; in mark-recapture studies adults were found 18 months after marking.

*Chelobasis perplexa* is known to feed on *Calathea insignis* Hort. & Bull. (Marantaceae) and *Heliconia imbricata* (Kuntze) Baker in Costa Rica (Maulik 1932). Strong and Wang (1977) and Auerbach and Strong (1981) reported *Heliconia latispatha* as a larval host plant. The biology of this species is similar to that of *Calathea bicolor*.

The tribe Alurnini consists of six genera and 29 species (Staines 2002b) and contains some of the largest chrysomelids (25–45 mm). The tribe was revised by Fischer (1935) and I am in the process of revising it. Published life histories record various genera and species feeding on palms (Arecaceae) (Fischer 1935, Villacis Santos 1968, Macedo et al. 1994). Both Mesoamerican species, *Alurnus ornatus* Baly and *Alurnus salvini* Baly, have been collected at La Selva. *Alurnus salvini* is the more commonly collected species.

The New World tribe Prosopodontini contains the genus *Prosopodonta* Baly with 26 species found from Nicaragua to Ecuador (Staines 2002b). The genus is in need of revision.

Two species, *Prosopodonta distincta* (Baly) and *Prosopodonta dorsata* (Baly), have been collected at La Selva. McCoy (1984, 1985) reported *Prosopodonta dorsata* (as *Cheirispa*) adults and larvae feeding in accumulated leaf debris on the top of *Heliconia* leaves in Costa Rica and Ecuador. All other species of *Prosopodonta* have been reported as leaf-miners on various Arecaceae (Jolivet and Hawkeswood 1995). The photograph in McCoy (1984) is a *Prosopodonta* adult however the larval photograph does not resemble the known *Prosopodonta* larvae (Maulik 1931). All other species of *Prosopodonta* are associated with Arecaceae and I have only found *Prosopodonta dorsata* on unfurled palm fronds, never on *Heliconia*.

The tribe Sceloenoplini contains five genera and 299 species, with 154 species in the genus *Sceloenopla* Chevrolat (Staines 2002b). They are leaf-miners in a variety of plant families. This tribe is represented at La Selva by four genera and 20 species (see Table 1). There are 17 species of *Sceloenopla* known from La Selva. The biology is unknown for all species.

The tribe Chalepini consists of 55 genera and nearly 1000 species in the New World (Staines 2002b). Very few genera have been revised. All species studied are leaf-miners and appear to prefer dicots (Jolivet and Hawkeswood 1995). This tribe is represented at La Selva by 18 genera and 55 species (see Table 1). *Chalepus* is the most speciose genus with 14 species.

### Habitat specificity

Hispines can be found in most non-aquatic habitats at La Selva. There are 46 species which feed on rolled leaves and inflorences of Zingiberales. This one feeding guild accounts for 33% of the hispine species known from La Selva.

Most hispines species seem to be restricted to understory to mid-canopy level plants. Work on hispines has shown many species to be monophagous or narrowly oligophagous. These species are found mostly in relation to their host plants. Other hispines are broadly oligophagous or polyphagous and can be found in many habitats. A continuing problem in inventory work is determining if the specimen collected was actually on its host plant or was a transient. Much of the earlier literature on host associations does not specify whether the insect was feeding as an adult, was breeding on the plant, or merely resting on it.

Relatively few species have only been collected from canopy fogging but these have been almost always undescribed species. Some of these species may actually be breeding in epiphytes rather than the fogged tree. *Calliaspis rubra* (Olivier) and *Acentroptera pulchella* Guérin-Méneville have been associated with bromeliads (Bromeliaceae) in South America (Lowman et al. 1996; Mantovani et al. 2005).

### Biogeography

Most of the La Selva hispine fauna is closely related to South American species. Some species have distributions throughout the Neotropics such as *Aslamidium impurum* (Boheman), *Charistena ruficollis* (Fabricius), and *Imatidium thoracicum* Fabricius. However, the genera *Anisostena* Weise and *Glyphuroplata* Uhmann are most speciose in the Nearctic and the La Selva specimens are part of the southern extension of the genera (Staines 2002b). No La Selva hispines are exotic.

Many species appear to be Central American Atlantic lowland wet forest endemics but with congeners in South America. *Ocnosispa humerosa* Staines, *Sceloenopla bicolorata* Staines, *Sceloenopla bidentata* Staines, *Sceloenopla lutena* Staines, and *Sceloenopla nigropicta* Staines appear to fall into this category.

### Specimen identification

Of the 139 hispine species known from La Selva, 125 (89.9%) are described species with published names, one is a morphospecies which is known to be new, and 14 (11.2%) are morphospecies in groups whose taxonomy is too poorly known to determine whether they are new or not.

La Selva hispine species can be identified using the key to the genera in Staines (2002b). All genera and species of La Selva hispines are in the “hispines of La Selva” web site (http://viceroy.eeb.uconn.edu/ALAS/ALAS.html). This site includes a summary of hispines, species lists, keys to species, references to revisions and other taxonomic publications, and individual species accounts with images and natural history data.

### Suggestions for future Research

What do hispines eat? A little more than half (63.3%) of La Selva hispines have any host plant association. Many of these have only been noted as being collected on a plant rather than actually feeding on it (listed as adult on Table 1). Since hispines are intimately tied to their host plant, determining the food plant will give a much better picture of their distribution and abundance. Additional leaf-miner rearing work such as that of Hespenheide and Dang (1999) is needed to make larval host plant associations. Johnson (2004a, b), Johnson and Horvitz (2005), García-Robledo and Horvitz (2009, in press), and García-Robledo et al. (2010) worked on the biology and ecology of several *Cephaloleia* species at La Selva.

What is the biology and ecology of hispines? Very little work has been done on the biology and ecology of La Selva hispines. Kirkendall (1984) studied the mating behavior of the North American *Odontota dorsalis* (Thunberg). Eberhard (1994) mentioned a hispine in his study of insect and spider courtship behavior. Staines and Staines (2001) and Flowers and Hanson (2003) suggested chrysomelids as potential indicator species assemblages for natural area monitoring. Farrell and Erwin (1988) showed that chrysomelids are a good indicator of local species richness. None of these ideas have been applied to hispines at La Selva.

What are the hispine host plant interactions? Strauss (1988) demonstrated that chrysomelids are a useful group for studing these interactions. Some work by Strong and his students (Strong 1977a, 1977b, 1981, 1982a, 1982b, Strong and Wang 1977), Horvitz and Schemske (2002), García-Robledo and Horvitz (2009, in press), and García-Robledo et al. (2010) have added to our knowledge of this but much remains to be done.

How do pathogens, predators, and parasitoids influence hispine populations? Hispines are parasitized by various wasps and flies (Cox 1994) and mites (Santiago-Blay and Fain 1994). They also have a few recorded predators (Cox 1996) and pathogens (Balazuc 1988, Hazarika and Puzari 1990). Memmott and Godfray (1993), Memmott et al. (1993), and Lewis et al. (2002) developed food and parasitism webs for some hispine species. A great deal of work needs to be done on how these organisms interact and what effect they have on hispine populations and distribution.

How do hispine populations and distributions change over time? Staines (2004b) studied the changes in chrysomelid populations over time on Plummers Island, Maryland. With the baseline inventory data and local knowledge at La Selva, a similar project could be started.
